# A yeast cell cycle model integrating stress, signaling, and physiology

**DOI:** 10.1093/femsyr/foac026

**Published:** 2022-05-25

**Authors:** Stephan O Adler, Thomas W Spiesser, Friedemann Uschner, Ulrike Münzner, Jens Hahn, Marcus Krantz, Edda Klipp

**Affiliations:** Institute of Biology, Theoretical Biophysics, Humboldt-Universität zu Berlin, Invalidenstr. 42, 10115 Berlin, Germany; Institute of Biology, Theoretical Biophysics, Humboldt-Universität zu Berlin, Invalidenstr. 42, 10115 Berlin, Germany; Institute of Biology, Theoretical Biophysics, Humboldt-Universität zu Berlin, Invalidenstr. 42, 10115 Berlin, Germany; Institute for Medical Informatics and Biometry, Technische Universität Dresden, Fetscherstr. 74, 01307 Dresden, Sachsen, Germany; Institute of Biology, Theoretical Biophysics, Humboldt-Universität zu Berlin, Invalidenstr. 42, 10115 Berlin, Germany; Laboratory of Cell Systems, Institute for Protein Research, Osaka University, 3-2 Yamadaoka, 565-0871, Suita, Osaka, Japan; Institute of Biology, Theoretical Biophysics, Humboldt-Universität zu Berlin, Invalidenstr. 42, 10115 Berlin, Germany; Institute of Biology, Theoretical Biophysics, Humboldt-Universität zu Berlin, Invalidenstr. 42, 10115 Berlin, Germany; Institute of Biology, Theoretical Biophysics, Humboldt-Universität zu Berlin, Invalidenstr. 42, 10115 Berlin, Germany

**Keywords:** cell cycle, mathematical modeling, cyclins, pheromone, osmotic stress, oscillations

## Abstract

The cell division cycle in eukaryotic cells is a series of highly coordinated molecular interactions that ensure that cell growth, duplication of genetic material, and actual cell division are precisely orchestrated to give rise to two viable progeny cells. Moreover, the cell cycle machinery is responsible for incorporating information about external cues or internal processes that the cell must keep track of to ensure a coordinated, timely progression of all related processes. This is most pronounced in multicellular organisms, but also a cardinal feature in model organisms such as baker's yeast. The complex and integrative behavior is difficult to grasp and requires mathematical modeling to fully understand the quantitative interplay of the single components within the entire system. Here, we present a self-oscillating mathematical model of the yeast cell cycle that comprises all major cyclins and their main regulators. Furthermore, it accounts for the regulation of the cell cycle machinery by a series of external stimuli such as mating pheromones and changes in osmotic pressure or nutrient quality. We demonstrate how the external perturbations modify the dynamics of cell cycle components and how the cell cycle resumes after adaptation to or relief from stress.

## Teaser

The yeast cell cycle has a tight control system and responds to external perturbations, which are considered here in a quantitative model.

## Introduction

The cell cycle machinery coordinates all processes that are required for a cell to duplicate and ensure faithful inheritance of all its critical components. Therefore, it is per definition deeply entangled with nearly all physiological processes that happen within and around a cell. Yet, the cell cycle machinery itself is already a large and complex network of many interacting partners with regulation spanning many levels, including transcriptional and posttranslational control as well as stoichiometric inhibition or activation of protein function (Enserink and Kolodner 2010). It is exactly for this reason that research has focused on understanding the cell cycle network itself in isolation. To this end, it was crucial to define the single interactions between specific cell cycle components to map out the network architecture. Specifically, mutant phenotypes have been exploited to understand the role of, and the type of interactions between, individual components (Hartwell *et al*. 1974). With this knowledge in place, the systems biology approach could be applied to try and predict the dynamics of multiple interacting components, sub-networks, simplified versions of the network, or even the entire network itself (Barberis *et al*. 2007, Chen *et al*. 2000, Goldbeter 1991, Kaizu *et al*. 2010, Münzner *et al*. 2019). The logical next step is to shift the focus from understanding the cell cycle network in isolation to integrating it into the larger physiological context of the cell. While the main purpose of the cell cycle machinery appears to be the coordination of the cell division events, this coordination is also heavily regulated by internal and external cues. Thus, it must be considered as part of a greater network: Its interfaces to other processes must be defined and its action must be evaluated within the context of these other processes, signals and conditions. Here, we aim to paint this bigger picture and illustrate it with a model of the cell division cycle that integrates information on the cellular state with environmental cues to make appropriate decisions to arrest or progress.

## Yeast cell cycle overview

The cell division cycle describes the life cycle of a single cell from one division to the next. It is divided into four phases based on observations of the DNA replication cycle. These phases are called synthesis (S) phase, in which DNA is replicated, and mitosis (M), in which the chromosomes are separated between the progeny cells. The S and M phases are interspaced by two gap phases (G_1_ and G_2_) in which the cell primarily grows. Various checkpoints monitor transitions between the phases controlling that the processes of the previous phase have been completed and, otherwise, arrest cell cycle until the requirements are met (Hartwell and Weinert 1989). In *Saccharomyces cerevisiae*, the main growth control of the cell division cycle occurs before the entry into S phase (Johnston *et al*. 1977). This checkpoint is called START and monitors whether the cell has the resources required to complete a new round of replication and cell division (Hartwell *et al*. 1974). Once past START, cells commence to replicate their DNA, duplicate the spindle pole body (yeast centrosomes) and start growing a new daughter cell as a bud. These processes are tightly regulated and monitored for completion, such that wild type cells only enter mitosis with fully replicated DNA, two spindle pole bodies, and a bud large enough to house the daughter nucleus as well as all other cellular structures required. Last but not least, the cell prevents the separation of its chromosomes until they are adequately aligned at the metaphase plate. This measure is taken to prevent untimely or improper distribution of the genomic material between the two newly emerging cells.

**Figure 1.**
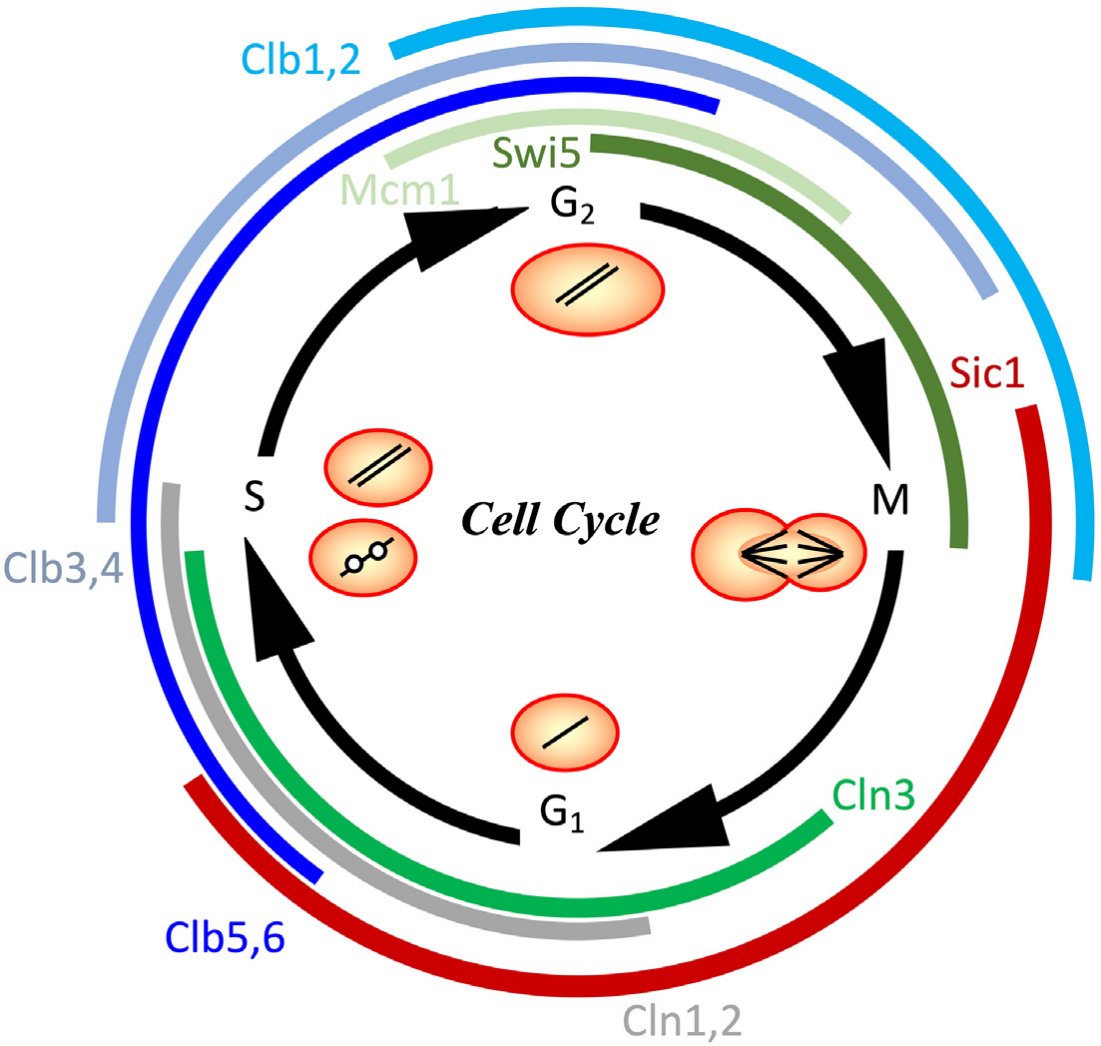
*Schematic overview of the temporal succession of events in the S. cerevisiae cell cycle*. The central circle symbolizes the cell with different copy numbers of DNA in different phases (one copy in G_1_, duplication in S, two copies in G_2_ and distribution to the progeny cells in M). The outer circle indicates the periods of expression of different cell cycle regulators as described by the computational model.

In order to understand the regulation and adaptation of the cell cycle upon internal and environmental perturbations, one first has to review the normal progression of the cell cycle and its contributing elements. The core regulatory mechanism of the cell division cycle is the sequential accumulation and destruction of cyclins (overview in Fig. 1). The cyclins bind to and activate the constitutively expressed cyclin dependent kinase Cdc28 (Cdk1). Cdc28 interacts with nine cyclins that are expressed in alternate phases of the cell division cycle (Enserink and Kolodner 2010). Three of the cyclins are G_1_ cyclins: Cln1, Cln2, Cln3, and six are B-type cyclins: Clb1, Clb2, Clb3, Clb4, Clb5, Clb6 (Pines 1995). With the exception of Cln3, the cyclins seem to function in pairs (Kellis *et al*. 2004, Wolfe and Shields 1997), such that the functions of the paralogs Cln1/2, Clb1/2, Clb3/4 and Clb5/6 are roughly equivalent.

Starting in early G_1_, Cln3 is the first cyclin to be expressed and associated with Cdc28 (Nash *et al*.^1988^). The Cdc28-Cln3 complex phosphorylates the transcriptional repressor Whi5 (De Bruin *et al*. 2004). In early G_1_, Whi5 is bound to the transcription factor complex Swi4-Swi6 (SBF) to inhibit its activity (Jorgensen *et al*. 2002). Upon phosphorylation, Whi5 is excluded from the nucleus. Activation of SBF and another prominent transcription factor complex called MBF (Mbp1-Swi6) at the end of the G_1_ phase leads to the transcriptional activation of more than 200 genes, to trigger downstream events such as budding and DNA replication (Spellman *et al*. 1998). Functionally, SBF and MBF targets are quite different (Wittenberg and Reed 2005). MBF activates expression of genes coding for proteins that activate DNA replication, such as *POL2*, *CLB5* and *CLB6*. SBF on the other hand activates genes that drive cell morphogenesis or the spindle pole duplication (Wittenberg and Reed 2005). *CLN1* and *CLN2* are crucial targets of SBF (Wittenberg and Reed 2005). Cln1 and Cln2 bind to and activate Cdc28, further increasing the phosphorylation of Whi5, and hence closing the positive feedback loop that stabilizes Cdc28 activity at the end of G_1_ (Skotheim *et al*. 2008).

The B-type cyclins regulate DNA replication and the entry into mitosis. To prevent premature DNA replication, the activity of the first B-type cyclins to be expressed, Clb5/6, is inhibited through high levels of the cyclin dependent kinase inhibitor (CKI) Sic1 during G_1_. Sic1 is a stoichiometric inhibitor of Cdc28 when the latter is bound to one of the B-type cyclins (Mendenhall 1993). When the critical Cdc28 activity near the end of G_1_ stabilizes, Sic1 is hyperphosphorylated by Cdc28-Cln1/2 forcing it to release Cdc28-Clb5/6 and targeting Sic1 for degradation (Kõivomägi *et al*. 2011, Verma *et al*. 1997). Cdc28-Clb5/6 in turn can also phosphorylate Sic1, which forms another positive feedback loop. Cdc28-Clb5/6 activates DNA replication, which per definition marks the S phase of the cell division cycle. The cyclins Clb3 and Clb4 are expressed approximately during mid-S phase, but their function remains largely unclear. Near the end of S phase, the transcription factor Mcm1 is expressed, and recruits the forkhead transcription factor Fkh2 and the co-activator Ndd1 to the *CLB2* promoter. Cdc28-Clb1/2 phosphorylates Ndd1, which is important for its recruitment to the *CLB2* promoter (Darieva *et al*. 2003; Reynolds *et al*. 2003), constituting another positive feedback loop.

The final B-type cyclins, Clb1/2, are required for mitotic entry and the isotropic switch. Cdc28-Clb1/2 regulates mitotic spindle elongation (Liang *et al*. 2013) and spindle pole body separation (Fitch *et al*. 1992). Cdc28-Clb1/2 activity is antagonized by the Swe1 kinase, which phosphorylates Cdc28 to inactivate it. Reciprocally, Cdc28-Clb1/2 phosphorylates Swe1, priming it for degradation after ubiquitination by the Anaphase Promoting Complex (APC). The tyrosine phosphatase Mih1 reverses the inhibitory phosphorylation on Cdc28. This causes Swe1 to be hyperphosphorylated by Cdc28, which allows for the full activation of the Cdc28-Clb1/2 complex. Cdc28-Clb1/2 also inactivates SBF (Amon *et al*. 1993, Koch *et al*. 1996), causing a loss of Cln1 and Cln2 from the cell.

For cells to exit Mitosis, Cdc28 activity must be reduced drastically. The cell achieves this with three synergistic strategies; by activation of the APC/C ubiquitin ligase, by activation of the Cdc14 phosphatase, and by expression of the CKI Sic1. Cdc20 is activated at the metaphase–anaphase transition, and associates with APC to target Clb1-6 for destruction (Lim *et al*. 1998, Shirayama *et al*. 1999, Yeong *et al*. 2000). However, the complete removal of the Clb1/2 protein from the cell requires activation of Cdh1. Cdh1 is another subunit of APC. The Cdc14 phosphatase reverses the Cdc28 mediated phosphorylations, and is responsible for the activation of Cdh1 through dephosphorylation (Jaspersen *et al*. 1999). Cdc14 also dephosphorylates and activates Swi5, a transcription factor for *SIC1*, and dephosphorylates Sic1 itself to prevent it from being degraded (Visinti *et al*. 1998). Sic1 can inhibit residual Cdc28-Clb kinase activity. During most of the cell cycle, Cdc14 is sequestered in the nucleolus, bound to Net1. The Cdc14-Net1 complex is also known as the regulator of nucleolar silencing and telophase (RENT) (Shou *et al*. 1999, Visintin *et al*. 1999). Cdc28-Clb1/2 phosphorylates Net1, causing a Cdc14 release from the inactive RENT complex. However, sustained activity of Cdc14 requires release from the RENT complex via Net1 phosphorylation by the Mitotic Exit Network (MEN), which does not happen until the daughterbound spindle pole body has entered the bud, thus preventing premature mitotic exit. Whi5 is dephosphorylated by Cdc14 to reset the conditions for a new G_1_ phase in the subsequent cell division cycle

## Yeast cell cycle models

The intriguing pattern of cyclin oscillations observed during the cell cycle has provoked modeling efforts from very early on. The aim was to understand which reaction network can bring about the oscillatory behavior. The discovery of the role of Cdc28 was worth a Nobel Prize for Lee Hartwell, Tim Hunt, and Paul Nurse in 2001. On this basis, Albert Goldbeter formulated the first mathematical model of the cell cycle (Goldbeter 1991). It contains only three species: a cyclin C, which can be produced and degraded, a kinase M which gets activated by binding of C and the protease X that is phosphorylated by M. The active protease then quickly degrades C. This negative feedback loop with delay and nonlinear kinetics leads to stable oscillations in a large part of the parameter space. After the ability to oscillate was demonstrated, later models incorporated more details about the individual cyclins and their interaction with other regulatory compounds, such as in the elaborated networks introduced (Chen*et al*. 2000, 2004). These models have been challenged by testing their predictive power and accuracy (Cross *et al*. 2002). A series of models has also focused on other critical aspects of the cell cycle machinery such as the regulation of mitotic exit by the interplay of molecular antagonists (Ciliberto *et al*. 2005), the role of feedbacks for the irreversibility of cell cycle transitions (He *et al*. 2011, Novak *et al*. 2007), size determination critical for entrance into S phase (Barberis *et al*. 2007), the role of multiple phosphorylations (Kapuy *et al*. 2009), the impact of osmotic stress on G_1_/S transition (Adrover *et al*. 2011) or cell cycle duration (Radmaneshfar *et al*. 2013), the entrainment of mammalian cell cycle by the circadian clock (Gérard and Goldbeter 2012), the role of specific transcription factors on cell cycle timing (Linke *et al*. 2013), the issue of cell cycle duration and cell size control in non-synchronized yeast population (Spiesser *et al*. 2012), the importance of the localization of specific cell cycle components (Spiesser *et al*. 2015), and many more. All of these models are useful in their own right, focusing on specific aspects of the cell cycle to highlight their influence or particular dynamics while per definition disregarding others for the sake of abstraction and reduction of complexity. Here, we build on these models to create a model that is flexible enough to serve as a scaffold for the integration into a larger cellular context. Specifically, it is flexible enough for modular extension, detailed enough to plug in other cellular components, simple enough to comprehend and work with, and available in a standard modeling format.

## Cell cycle in physiological context

Since we shift the focus towards the behavior of cell cycle progression in a larger physiological context, it is necessary to define the interfaces via which external signals are integrated into the cell cycle machinery. It is critical for cells to sense and react to environmental conditions, in order to maintain optimal proliferation and even ensure survival. To that purpose, yeast cells employ signal transduction pathways that relay information to steer adaptation programs, but also heavily interfere in the progression of the cell cycle. For example, mitogen-activated protein kinase (MAPK) cascades are employed for this task. Many interactions between components of signaling pathways and the cell cycle machinery have been described, e.g. the cell wall integrity pathway (CWI) (Levin 2005); the high osmolarity glycerol pathway (HOG) (Hohmann 2002, Adrover*et al*. 2011, Clotet *et al*. 2006) or the filamentous growth pathway (Gimeno *et al*. 1992, Kron *et al*. 1994, Rua *et al*. 2001). However, for the scope of this work, we focus on two of the best studied: the impact of the osmotic stress response and the pheromone signaling pathways on cell cycle progression. To this end, we incorporate interfaces for these signaling pathways. These exemplify the extendibility of our model and how it can be integrated with further pathways and/or in a larger context. Below, we introduce the relevant processes and how they interact with the cell cycle machinery.

Growth is the most important determinant of cell cycle progression. The growth rate of unicellular organisms is determined by nutrient availability and influences cell size, ribosome content and metabolic efficiency (Crebelli *et al*. 1991, Molenaar *et al*. 2009, Scott and Hwa 2011). Due to the resulting changes in protein content and synthesis rate, the cell cycle duration is responsive to nutritional conditions. It is slower in poor media with, e.g. ethanol as energy source, and faster in rich fermentable media with, e.g. glucose as energy source. However, the growth rate is further regulated by signaling pathways in addition to these direct effects of nutrition. Some signaling pathways that sense changes in nutrient availability and regulate metabolic processes, such as the TOR (Target of rapamycin), Snf1, and the PKA (Protein kinase A) pathways, also directly regulate cell cycle progression. Other signaling pathways respond to other types of perturbations, such as changes in osmolarity, temperature, or the presence of mating pheromones. Here, we focus on two signaling pathways that respond to such perturbations; the pheromone pathway as well as the High Osmolarity Glycerol (HOG) pathway.

The pheromone pathway is essential for mating. In G_1_ phase, haploid cells can suspend the mitotic cell cycle and instead mate with a compatible partner (Herskowitz 1988). This enables the cells to form a diploid cell, which in turn can undergo sporulation under challenging environmental conditions to generate haploid cells. Yet for mating to be possible, haploid yeast cells need to communicate and synchronize their cell cycles. They do this by producing and secreting either alpha- or a-factor, i.e. pheromones that indicate the presence of potential mating partners nearby and can be sensed by the complementary cell type (Arkowitz 2009, Merlini *et al*. 2013). This leads to formation of a protrusion called ‘shmoo’, with which the cell grows to close the distance to the mating partner, and ultimately to fuse the two cells, creating a single diploid cell that then can undergo mitosis or meiosis and sporulation. However, the cell cycle in both mating partners needs to be stopped first to prevent them from entering S phase. Without this crucial synchronization, mating might not be possible at all or only at high risk. Yet, not only is this pheromone induced cell cycle arrest important for yeast populations in their natural habitats, but it has been widely used as cell cycle synchronization mechanism in experimental setups as well (Breeden 1997). Using haploid MATa yeast strains that are unable to switch their mating type is one of the main techniques to synchronize the cell division cycle state within a cell culture, which can be studied after release from the pheromone treatment. We introduced this mechanism into our model by connecting the cell cycle module and the yeast pheromone response pathway with a structural interface. This includes the protein kinase Fus3 as the inducing activator as well as Far1 for facilitating the main response mechanisms leading to the arrest. Both Fus3 and Far1 become activated by the pheromone signaling pathway (Kofahl and Klipp 2004) that involves a G-protein coupled receptor and a MAP kinase cascade as well as several negative feedback loops to relay the external information on pheromone presence.

Not only do yeast cells have to communicate with one another in order to ensure optimal proliferation, but also each cell must be able to react and adapt to environmental changes and challenges. As with pheromone sensing, a network of signaling pathways that coordinate adaptational programs in yeast mediates those stress responses. In the case of increased osmolarity (e.g. high salt or sugar concentrations in the environment), the well-studied High Osmolarity Glycerol (HOG) pathway mainly coordinates the adaptation to increased osmolarity (Adrover*et al*. 2011, Klipp *et al*. 2005, Petelenz-Kurdziel *et al*. 2013). This pathway reacts on changes in osmolarity by activating its name-giving MAP kinase Hog1 via a cascade. Once activated, this kinase targets multiple transcription factors (e.g. Hot1, Sko1, and Smp1) as well as metabolic enzymes (e.g. Pfk26) that subsequently trigger several responses to adapt the cell both transiently as well as in a long term to the stress. The effects of high osmolarity include outflow of water, pressure on the cell wall (changes in turgor), changes in concentrations and ionic strength. For the cell's survival it is important that the adaptation to this stress is prioritized and proliferation is stopped in a timely manner.

## A dynamic cell cycle model setup for integration into a larger physiological context

Below, we present a new cell cycle model that incorporates all these features. It works autonomously, is able to accommodate the interactions with signaling pathways, and to react appropriately to environmental or internal stimuli. In particular, we show that the implemented motif for combined action of Fus3 and Far1 prevents the cell from entering S phase for as long as pheromone signaling is active. The arrest is lifted once the pheromone signal ceases. Thus, our model captures the synchronizing biological function seen in yeast cell populations. Also, we analyze cell cycle progression under the influence of osmotic stress. To this end, we introduced molecular interactions between the activated HOG pathway output, namely double phosphorylated Hog1, and various cell cycle components. Our implementation enabled us to reproduce *in vivo* responses, i.e. arresting the cell cycle in different phases and reentering cell cycle progression upon stress relief. This can be seen as a prototype for many eukaryotic stress responses as this MAP kinase motif has been conserved over a large variety of organisms. Finally, we explore the impact of variations in nutrient supply via modification of the protein synthesis capacity of the cell. We show that, in our model, the cell cycle duration is responsive to the nutritional condition. Taken together, we showcase a new model of the cell division cycle and demonstrate how it can be part of a greater cellular network. The model integrates information on the cellular state with environmental cues to make appropriate decisions to slow down, speed up, or arrest the cell division cycle. This marks it a valuable asset to build upon in future applications on the way to ever more complex and comprehensive cell models.

### Model details

**Figure 2.**
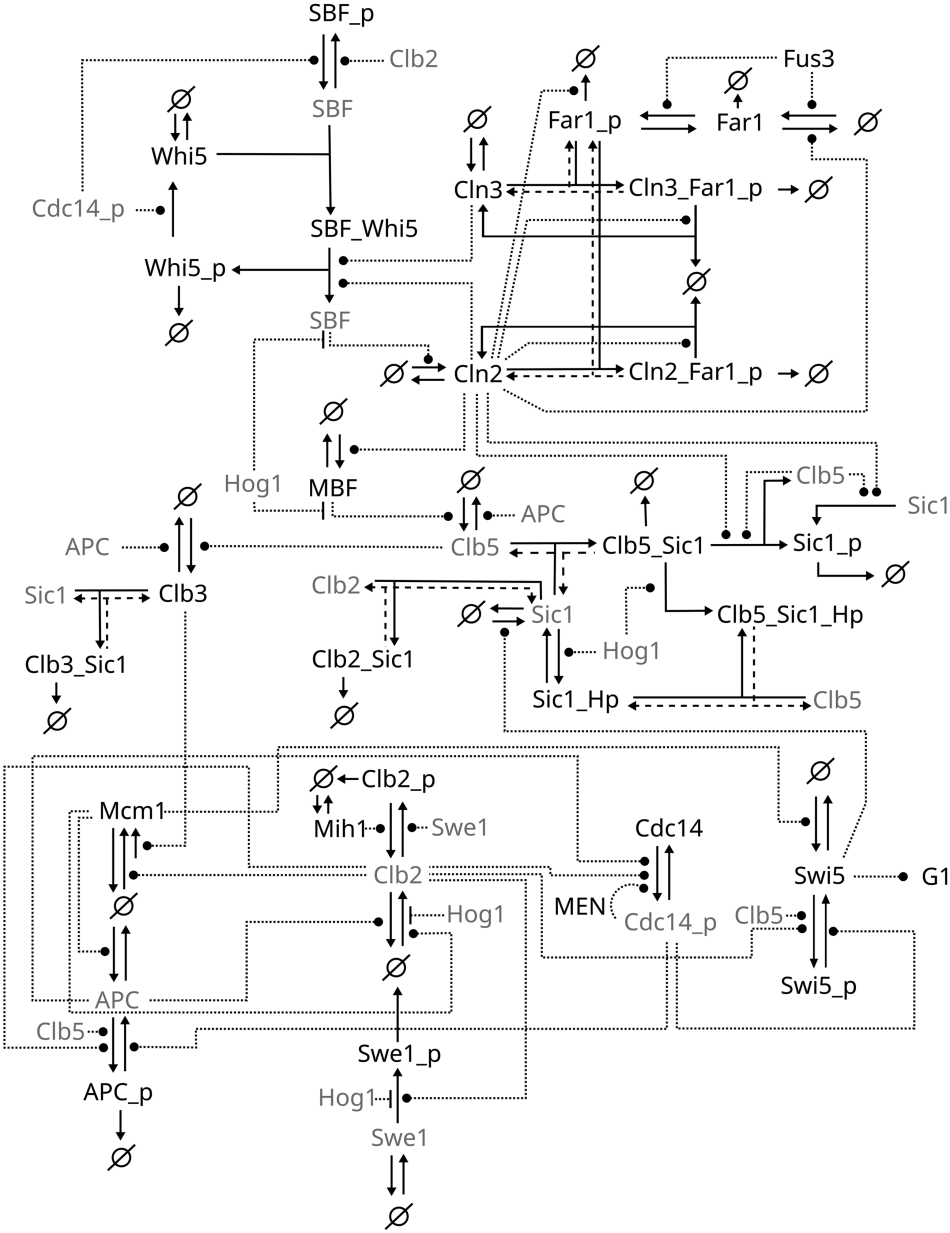
*Schematic of the model network structure*. Arrows indicate reactions; dotted lines represent modifications (bullets: positive, dash: negative). The ‘_p‘ flag indicates phosphorylation. Sic1 phosphorylation by Hog1 is indicated with a special ‘_Hp‘ flag to distinguish between the Cdc28 and Hog1 phosphorylation. To increase the readability of the schema and reduce the number of very long arrows, some model species (e.g. Clb2) appear more than once in the figure. These species are shaded in gray. Some backwards reactions are shown with dashed lines only to improve readability. Cyclin paralogs are represented by what we considered the major contributor to the indicated reactions, and Cdc28 is omitted for sake of brevity, such that Cln2 stands for Cdc28-Cln1/2, Clb5 for Cdc28-Clb5/6, Clb3 for Cdc28-Clb3/4, and Clb2 for Cdc28-Clb1/2.

We opted to create a representation of the cell cycle that would be comprehensible, but at the same time detailed enough to serve as a scaffold for the integration of various signals and conditions to acknowledge the larger cellular context. To this end, we reduced and simplified it to a point where the model is still manageable, yet the basic cell cycle mechanisms are in place, especially regarding checkpoints that are invoked by the interfaces with surrounding cellular processes we consider here. Thus, the model has different level of detail for different parts of the network (Fig. 2). The implementation details of the cell cycle interfaces with the pheromone signaling pathway and osmotic stress response pathway are described in their respective sections. In the following, we focus on the implementation of the core cell cycle mechanisms and their implications for the systems dynamics.

### G_1_

**Figure 3.**
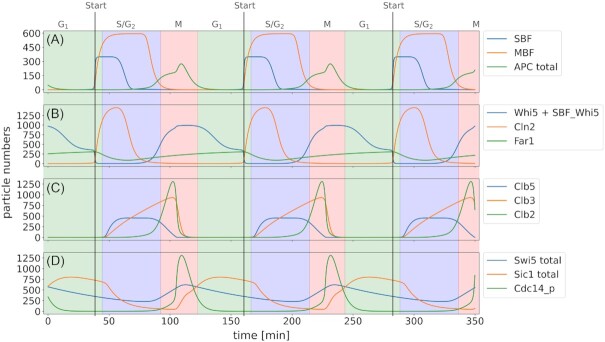
*Simulation time courses of key species in the model show oscillating behavior*. The *total* annotation in the legend means that the total amount of correspondent molecules in the system, including all their complexes and states (e.g. Sic1_total = Sic1 + Sic1_p + Sic1_Hp + Clb5_Sic1 + Clb5_Sic1_Hp + Clb3_Sic1 + Clb2_Sic1), are shown in a single trajectory. Other names refer to the actual model species without complexes or modifications. For Whi5 the time course of nuclear Whi5 (Whi5 + SBF_Whi5) is given. The shaded fields indicate different cell cycle phases: G_1–_green, S/G_2–_blue, M—red. START is the point of commitment to another round of DNA replication. Without making use of events for cell cycle progression, the model is able to produce robust consecutive cell cycles.

To account for the early events in a cell's life cycle, we include components of the highly sophisticated G_1_ network as described in the introduction. The G_1_ network communicates external and internal cues to adjust the timing of the START transition to external growth conditions and inner cellular physiology. As expected, simulations of the model trajectories show that in early G_1_, nuclear Whi5 levels are high, thus repressing SBF activity (Fig. 3). Sic1 and Far1 keep Cdc28 activity low. Cln3 is the only cyclin expressed in early G_1_, and Cdc28-Cln3 continuously phosphorylates Whi5. Phosphorylated Whi5 releases SBF on the *CLN1/2* target promoter and is excluded from the nucleus. *CLN1/2* is then expressed, which causes the Cdc28-Cln1/2 feedback to kick in to fully phosphorylate Whi5 and ensure the START transition. It was suggested that Cdc28-Cln1/2 activity is required to activate MBF in a Whi5 independent mechanism (Wittenberg and Reed 2005), which is why Cdc28-Cln1/2 directly activates MBF in the model (Fig. 2). MBF activity triggers *CLB5/6* expression. Although we do not model DNA replication here, Cdc28-Cln5/6 would eventually induce it, which is why this would be the place to incorporate a DNA replication model. We consider SBF dependent transcription and Whi5 nuclear exclusion as a mark for the START transition and half-maximal Cdc28-Clb5/6 (total) activity as a mark for the G_1_/S transition (Fig. 3).

### S

In S phase, the simulated Cdc28 activity is high through high levels of Cln1/2, Clb5/6 (Fig. 3) and, from mid-S phase on, Clb3/4. While it is known that ongoing DNA replication maintains S-phase gene expression (through inhibition of the Nrm1 repressor) and inhibits the G_2_/M transition (through inhibition of the Ndd1 activator and stabilisation of the CDK tyrosine kinase Swe1) (Münzner *et al*. 2019), we needed a different solution as the model does not include DNA replication and Rad53/Mec1 signalling. Instead, we implement an alternative mechanism of step-wise activation of Clb1/2 by Clb3/4, and Clb3/4 by Clb5/6, as proposed by (Mondeel *et al*. 2020, Linke *et al*. 2017, Barberis 2021a,b). Hence, we implemented a timer for the length of S phase through a cyclin cascade. While this implementation cannot account for massive delays in DNA-replication, we deem it a suitable compromise between accuracy and tractability of the model.

### G_2_

Under normal conditions, G_2_ is rather short in *S. cerevisiae* and so it is in the model. We simplified the mechanism of *CLB1/2* expression in the model: Mcm1 directly leads to *CLB1/2* expression without cofactors and Clb1/2 positively regulates its own gene expression by also directly activating Mcm1, which results in a positive feedback loop (Amon *et al*. 1993, Darieva *et al*. 2003, Reynolds *et al*. 2003). Concurrently, Mcm1 induces expression of *CDC20*, which binds and activates the APC. In the model, this is implemented as a direct activation of APC by Mcm1. Swe1 and Cdc28-Clb1/2 phosphorylate and inactivate one another. In agreement with the experimental evidence, the Mih1 phosphatase helps to tip the scales towards Cdc28-Clb1/2, eventually removing the inhibitory phosphorylation, seen as a rise in Cdc28-Clb1/2 (Fig. 3). Here, we consider half-max Cdc28-Clb1/2 levels as a mark for the G_2_/M transition.

### M

When Cdc28-Clb1/2 activity has stabilized, the cell enters M phase. Cdc28-Clb1/2 phosphorylates SBF, which causes a loss of Cln1/2 from the cell, and Net1, which triggers release of Cdc14 from its inactive state (Fig. 3). Evidence suggests that a second surge of Cdc14 activity is triggered through release from the RENT complex via Net1 phosphorylation by the MEN, which in turn is activated by Cdc14, which is another positive feedback loop (Bardin *et al*. 2003). We implemented a highly simplified version of this positive feedback loop so that, in the model, Cdc14 positively regulates its own activity (Fig. 2). In a living cell, Cdc14 dephosphorylates Cdh1, which binds and activates APC. Active APC leads to degradation of Securin, which causes a release of the Separase protein. Separase in turn inhibits the PP2A phosphatase, which can then no longer dephosphorylate Net1, leading to less inactive RENT complex and ultimately to more active Cdc14 (Queralt *et al*. 2006). In the model, we do not distinguish between APC bound to Cdh1 or Cdc20, but consider a bound form as active APC and the unbound form as inactive. Also the process of Securin degradation is not represented in the model. Instead, we implemented it as a direct activation of APC on Cdc14 (Fig. 2). Cdc14 and APC counteract the Cdc28-Clb5/6 and Cdc28-Clb1/2 activity in mainly two ways. On the one hand, APC targets them directly to mark them for degradation, and, on the other hand, Cdc14 activates the transcription factor Swi5 that induces Sic1 expression. Sic1 abolishes residual Cdc28 activity through stoichiometric inhibition of any remaining Cdc28-Clb5/6 and Cdc28-Clb1/2, and the *in silico* cell is ready for a new G_1_ phase (Fig. 3).

### ODE model

**Figure 4.**
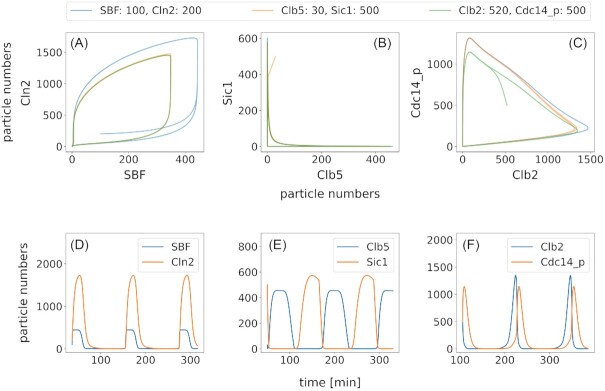
*Representation of the oscillatory behavior of the model*. Phase space examples of different mechanistic couples. Panel A shows trajectories of Cln2 and its corresponding transcription factor complex SBF for different initial conditions. The numbers in the legend indicate initial conditions that deviate from other simulations. Noticeably, the trajectories find different attractors for the different conditions. This is due to a change of total SBF in the system, that is neither produced nor degraded in the model, but changes its state. Despite this strong interference the system finds stable oscillations. Panel B gives an example for the antagonistic behaviour of the activator-inhibitor-couple Clb5 and Sic1. Whenever one of them is abundant, the other one is absent, sharing only a very small temporal overlap when both levels are very low. This is when they can be found in complex. Panel C shows another activator-inhibitor-couple that share a more pronounced overlap, both peaking in M phase. Like SBF, Cdc14 p is part of a fixed pool (Cdc14 and Cdc14 p), so changing its initial conditions and therefore the pool leads to changed oscillations. All trajectories find a limit cycle as expected for oscillating systems. Panels D, E, and F show respective time course examples for comparsion.

The model is implemented as a system of 33 ordinary differential equations (ODEs) with 111 parameters (Tables S1 and S2). It is implemented in the Systems Biology Markup Language (SBML) and is available in the supplementary data (File S1). The cell cycle part of the model is implemented without events. However, we use events to turn pheromone signaling or osmotic stress on and off. The cell cycle duration of our reference condition, i.e. the standard parameter set (Table S2) without any stress, is ∼122 minutes. Our implementation results in limit cycle oscillations, as shown in Fig.4, with a representation of gradients provided in Figs S3 and S4 and a UMAP projection (McInnes *et al*. 2018) of the full dynamic behavior in Fig. S5.

### Parameter adjustment

All parameters were adjusted with respect to cell cycle phase duration (Skotheim *et al*. 2008, Ferrezuelo *et al*. 2012, Di Talia *et al*. 2007), as well as particle numbers (’*S.cerevisiae—*Whole organism (integrated)’ data set from the Protein Abundances Across Organisms database (PaxDB) (Wang *et al*. 2015)). The adjustment was achieved manually in an iterative process. To this end, first a parameter set was identified that showed stable cycling. Then the cycling was adjusted to result in realistic cell cycle phase durations. To adapt the particle numbers the model was run over several cycles until a stable average amount of all involved molecules was reached over time. These amounts were then compared to the experimental data and parameters were changed if necessary to compensate deviations. This procedure was repeated until the experimentally obtained cell cycle phase durations were met and all particle numbers were within a margin of less than 60% relative deviation from the measurements. A comparison of these numbers is given in Table S3 and Fig. S1. To test the model for robustness against parameter changes, all parameters were increased and decreased by 10% of their original value, respectively. The resulting simulations were tested for stable cycling and the resulting changes in cell cycle duration were obtained. The results can be seen in Fig. S2.

## Model response to physiological perturbations

In the following, we describe how the model behaves when exposed to alpha factor treatment, high osmotic stress, and how the cell cycle duration is affected by the availability of nutrients.

### Alpha factor mediated arrest leads to cell cycle synchronization

**Figure 5.**
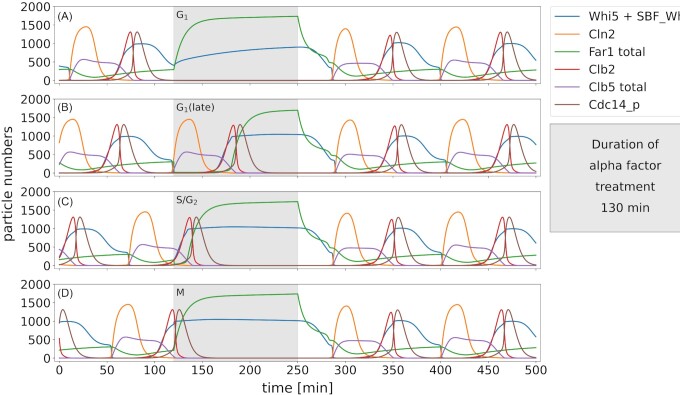
*Pheromone treatment leads to a synchronization of the cell cycle*. Unsynchronized cells are treated with pheromone for 130 minutes (grey shading), applied during different cell cycle phases (**A**: G_1_, **B**: late G_1_, **C**: S/G_2_ and **D**: M). G_1_: the cell arrests in G_1_. Late G_1_, S/G_2_ and M: The current cell cycle has already proceeded beyond the arrest point. The cycle finishes and the cell arrests in the subsequent G_1_ phase. All phases: Simultaneous release of pheromone treatment leads to a continuation of the cell cycle in a synchronized manner after a short period of adaptation.

Pheromone treatment leads to arrest before the G_1_/S transition. The arrest caused by the sensing of pheromone in the environment is mediated by the CKI Far1 (‘Factor ARrest’, (Chang and Herskowitz 1990)). The Fus3 MAP kinase cascade mediates an induction of Far1 transcription by activating the transcription factor Ste12 (Oehlen *et al*. 1996), thus elevating the concentration of Far1 upon pheromone exposure (Fig. 5). This is simplified in our model by omitting the intermediate step via Ste12 and making it a direct induction of the transcription rate for Far1 (Fig. 2). The key mechanism that leads to the arrest in G_1_ is a stabilizing phosphorylation of Far1 at Thr306 by Fus3 (Gartner *et al*. 1998), called Far1_p in the model. This form of Far1 associates with the kinase complexes Cdc28-Cln1/2 (Peter and Herskowitz 1994) and Cdc28-Cln3 (Jeoung *et al*. 1998, Tyers and Futcher 1993), inhibiting their activity and thus the further progression of the cell cycle. If the cell cycle has already passed START at the time of alpha-factor treatment, this mechanism is nonfunctional and the cell cycle will be completed before it arrests in the next G_1_ phase (Fig. 5B–D). This mechanism ensures that replication is properly executed and also provides the basis for cell synchronization, as it depicts a single unique point for the arrest—and hence release—throughout the subjected cell population.

Both Far1 as well as its stabilized form are subject to phosphorylation at the S87 residue by Cdc28-Cln1/2, triggering ubiquitination and proteasomal degradation (Henchoz *et al*. 1997). Release from alpha factor leads to a rapid recovery of Cdc28-Cln complex levels and thus, after a short adaptation period, a continued oscillation of the cell cycle (Fig. 5).

Cells released from alpha-factor are synchronized in their cell cycle, independent of the time point when the cell was treated with the pheromone (compare Fig.5A–D). This effect is observed in experiments and frequently exploited for cell cycle synchronization in haploid yeast populations.

### The model responds to Hog1-mediated osmotic stress with a cell cycle stage dependent program

**Figure 6.**
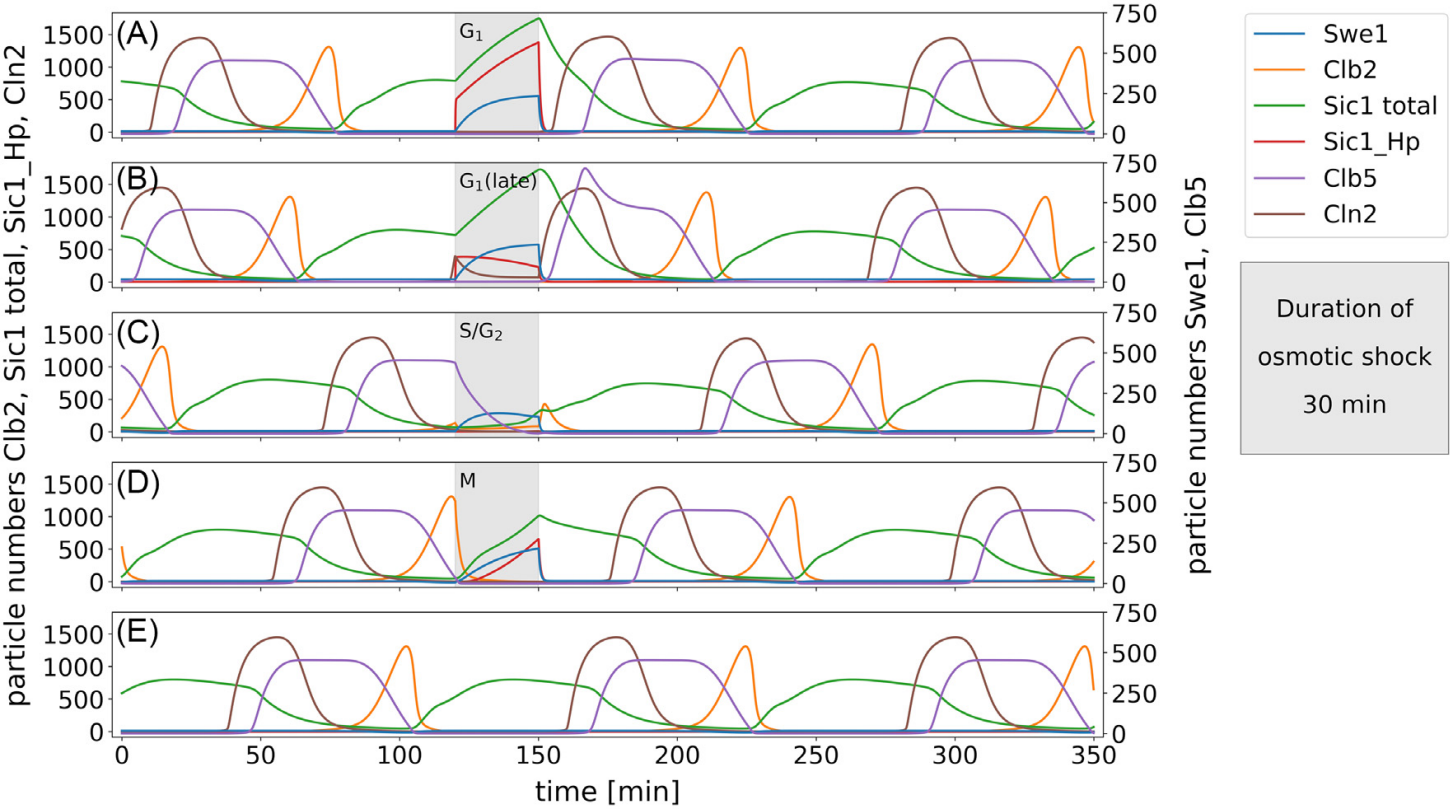
*Model response to osmotic stress depends on the cell cycle phase in which the stress appears*. Cells are subjected to osmotic stress for 30 minutes (grey shading) in different cell cycle phases (A: G_1_, B: late G_1_, C: S/G_2_ and D: M). Panel E shows the corresponding time courses of an unstressed cell. The arrest mechanisms differ, depending on which components are active in the respective cell cycle phase. The M phase is the exception where the cycle continues without arrest. After the stress is lifted, the oscillatory behavior of the cell cycle is re-established after a short adaptation period. Since Swe1 expression is not regulated in the model it also accumulates during osmotic stress in G_1_, which is not reported in the literature. However, since Clb2 is absent during G_1_ and Swe1 specifically acts on Clb1/2 bound Cdc28 , this accumulation does not cause any interference in the model.

**Figure 7.**
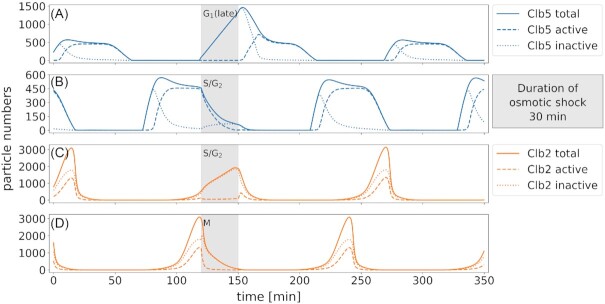
*Active and inactive forms of the cyclins Clb5/6 and Clb1/2 show the influence of cell cycle altering mechanisms in osmotic stress response*. Osmotic stress (grey shading) is applied to the cells during different cell cycle phases (A: late G_1_, B and C: S/G_2_ and D: M). Solid lines correspond to total particle numbers, dashed lines to active and dotted lines to inactive forms of the proteins Clb5/6 (blue) and Clb1/2 (yellow). Production of Clb5/6 is scaled down upon stress and, in addition, Clb5 is inactivated by forming a complex with (stabilized) Sic1 **(A**and**B)**. In S/G_2_ phase, Clb1/2 is inactivated through sustained levels of Swe1 **(C)**. Stressing the cell in M phase causes earlier mitotic exit **(D)**.

Budding yeast has developed adaptation programs to certain stresses in order to arrest the cell cycle, react to the stress appropriately and, upon successful adaptation, resume cell cycle progression. Osmotic stress induces activation of the HOG MAP kinase cascade, which ultimately leads to the activation of Hog1. The response on the level of cell cycle regulation is depicted in Fig.6. Due to the oscillatory nature of proteins during the cell cycle, stress responses are acted upon differently depending on the cell cycle phase. In unperturbed cells (Figs 3 and 6E), SBF and MBF regulated transcription is activated at the transition from G_1_ to S phase (Iyer *et al*. 2001). Upon Hog1 activation, however, the expression of *CLN1/2* and *CLB5/6* is downregulated (Adrover*et al*. 2011, Bellí *et al*. 2001, Wittenberg and Reed 2005), leading to an arrest in cell cycle progression. This effect is pronounced in late G_1_, where Cln1/2 and Clb5/6 are the prevalent drivers of cell cycle progression (Figs 6B and 7A, respectively). In addition, Hog1 prompts cell cycle arrest via a direct stabilizing phosphorylation of Sic1 at Thr173 (Escoté *et al*. 2004, Zapater *et al*. 2005). The Hog1 stabilized form of Sic1 is not subject to Cdc28 induced degradation and, hence, the levels of the Cdc28-Clb5/6-Sic1 complex rise, rendering Clb5/6 inactive during the stress and thus blocking cell cycle progression (Fig. 7A and B). In our model, the effect of osmotic stress is observable as delayed expression of Cln1/2, Clb5/6 (Fig. 6A) and Clb1/2 (Fig. 6A and B), and as inactivation of Clb1/2 (Figs 6B and 7C and D) and Clb5/6 (Fig. 7A and B) given that they were active at the time of stress. Stressing the cell during M phase can cause an early mitotic exit, depending on the progress of M phase (Radmaneshfar *et al*. 2013, Reiser *et al*. 2006) (Figs 6D and 7D). After the cell has adapted to the stress, cell cycle progression is resumed with a short adjustment period.

The G_2_ to M transition requires degradation of the Cdc28 kinase inhibitor Swe1. In the unperturbed cell cycle, Swe1 degradation requires its localization to the bud neck. This recruitment is mediated by Hsl7. Hsl7 forms a complex with Hsl1, which, in turn, is attached to septin. Export from the nucleus and tethering to the Hsl1/Hsl7 complex in the budneck primes Swe1 for phosphorylation by Clb2-Cdc28 and Cdc5 (Howell and Lew 2012). The kinase Cla4 also participates in Swe1 phosphorylation and targets it for ubiquitination (Yasutis and Kozminski 2013). Osmotic perturbations at this stage of the cell cycle lead to phosphorylation of Hsl1 by Hog1 forcing the dissociation of Hsl7 (Howell and Lew 2012). The disruption of the Hsl1/Hsl7 interaction interrupts Swe1 degradation, leading to a stabilization of Swe1 levels. The response to osmotic stress at the G_2_/M transition is implemented in a simplified manner: In the model, Swe1 phosphorylation is only mediated by Clb2. The stress induced Hog1 activity results in sustained Swe1 levels (Fig. 6A–D) and stalls cell cycle progression. This is achieved by rendering Clb1/2 inactive (Fig. 7C and D) or preventing its activation (Fig. 6A and B).

Additionally, Hog1 directly inhibits phosphorylation of Swe1 (Fig. 2), thus, the Hsl1/Hsl7 complex is omitted. Cell cycle progression resumes upon removal of the osmotic stress signal.

### Cell cycle duration depends on nutrient source

**Figure 8.**
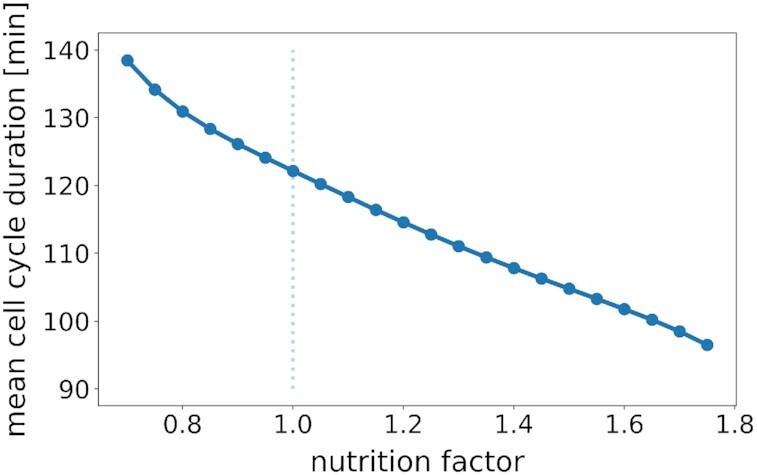
*Average cell cycle duration in response to changes of nutritional conditions*. All protein production rates were scaled with a factor (nutrition factor) to mimic different growth conditions where nutrition factor = 1.0 represents default, nutrition factor *< *1.0 poorer and nutrition factor *> *1.0 richer nutritional conditions. The average cell cycle duration of a single cell is shown, tracked over at least 10 subsequent cell cycles after a reasonable adaptation phase. The cell cycle duration decreases under richer and increases under poorer nutritional conditions.

It is well known that the cell cycle duration of *S. cerevisiae* changes with the nutritional condition the cells live in (Barford and Hall 1976, Di Talia *et al*. 2007, Ferrezuelo *et al*. 2012). There are signaling pathways, like PKA, that have been shown to directly modulate cell cycle progression (Baroni *et al*. 1994, Tokiwa *et al*. 1994). Others, like TOR, influence the growth or biosynthetic capacity of the cell (Jorgensen *et al*. 2002, 2004). In the previous paragraphs, we have shown that the model is able to respond to signaling pathways, which is why we chose to focus in this paragraph on the more generic effect of altering the growth capacity of the cell to observe the impact on the cell cycle. To mimic the effect of altered growth conditions, we systematically changed all production rates in the model simultaneously by multiplying them with a single factor (called nutrition factor). This simple procedure reflects a change in the biosynthetic capacity of the cell, be it through adapted ribosomal content, availability of building blocks and precursors or changed metabolic activity. The model responds to a change of the nutrition factor by adapting the average cell cycle duration (Fig. 8). The amount of change in cell cycle duration lies within a reasonable physiological range, with doubling times for fast growing cells of roughly 96 minutes (nutrition factor is 1.7) and for slow growing cells of about 138 minutes (nutrition factor is 0.7). The trend between these two extremes shows an almost linear response of average cell cycle duration due to nutrition factor changes (Fig. 8).

## Discussion

We present a model of the yeast cell division cycle that incorporates dynamics of the major cyclins, cyclin dependent kinase inhibitors, transcription factors, and other key players. The model structure is largely based on well-studied concepts of the cell cycle network (Barberis *et al*. 2007, Chen*et al*. 2000, 2004), but it is designed to serve as a scaffold model in a larger cellular context. This is why we put particular emphasis on balancing complexity and comprehensibility to ensure its ease of integration with other cellular components, such as signaling networks or metabolism. We exemplified this by (i) analyzing the impact of osmotic stress at different time points, (ii) following the effect of pheromone addition and removal, and (iii) analyzing the influence of the quality of nutrients on cell cycle duration. The model offers some important advantages: First, it describes the cell cycle dynamics with only ordinary equations, without artificial step functions or timing functions as used previously (Chen*et al*. 2000, 2004). As a result of this approach, the model exhibits certain key features that include (i) limit cycle oscillations purely based on the interaction network and its kinetics (Fig. 4), (ii) response to external and internal signaling and stress with the appropriate behavior (Figs 5 and 6), and (iii) quantitative changes of proteins and adapted timing for different nutritional conditions (Fig. 8). Second, while the model parameters have a large impact on the quantitative behavior, the model's key features are robust against parameter changes. Parameters have been determined to ensure protein concentrations to be in the order of magnitude of measured protein levels (Wang *et al*. 2015). Thus, our model is a prime candidate to investigate the regulation of the cell division cycle in regard to signaling pathways, stresses, or cues.

We used the model to specifically analyze the response to pheromone treatment. In accordance with experimental findings, the interaction of the pheromone pathway with the cell cycle was implemented via the modification of Far1 activity (Fig. 2). This implementation enabled us to analyze the effect of pheromone treatment at different points during the cell cycle (Fig. 5), and could serve to analyze different periods of pheromone treatment as well. Besides the obvious interest in contributing to the in-depth understanding of the interplay of the pheromone response and the cell cycle, there is another interesting aspect to be considered here. Pheromone treatment is a widely adopted laboratory technique to synchronize cell populations with regards to their cell cycle prior to population-based experiments. Our model nicely mirrors the synchronization effect, showing that cells stressed in different phases of their cell cycle are post-stress synchronized (Fig. 5). Interestingly, potential side effects of the pheromone treatment on cellular or population characteristics, such as the accumulation of larger cells or cells with a shmoo or cells with protein concentrations deviating from the normal behavior, are generally ignored in laboratory experiments. While the effect might well be negligible, it has never been quantified satisfactorily. This model—when combined with a growth module—could provide a starting point to unravel and quantify this effect. This might help bridging the gap between some of the single cell and population based data that so far must remain unexplained.

The osmotic shock response is the prime example used to study how single cells and cell populations cope with stress and recover from changes in their environment (Hohmann 2002, Adrover*et al*. 2011, Alexander *et al*. 2001, Bellí *et al*. 2001, Clotet and Posas 2007, Correia *et al*. 2010, Duch *et al*. 2013, Escoté *et al*. 2004, Migdal *et al*. 2008, Mizunuma *et al*. 2013, Nadal-Ribelles *et al*. 2014, Radmaneshfar *et al*. 2013, Waltermann *et al*. 2010, Yaakov *et al*. 2009). The application of osmotic stress has been implemented under consideration of the different ways of interaction that phosphorylated Hog1 can have with the cell cycle machinery (Fig. 2). We analyzed the effect of osmotic stress applied at different cell cycle stages and followed protein dynamics during and after stress (Fig. 6). Importantly, we find that the timing of osmotic stress is critical. When the osmotic stress occurs early in G_1_, cells arrest prior to Start, when the stress occurs later, however, cells pass into S phase and continue the cell cycle until reaching the next checkpoint. Interestingly, the model predicts that osmotic stress applied in M phase can lead to early mitotic exit (Radmaneshfar *et al*. 2013, Reiser *et al*. 2006) (Figs 6 & 7).

Growth is a fundamental property of life, which critically depends on the available nutrients. Therefore, we also analyzed the impact of change in the nutritional conditions on cell cycle progression. While the interfaces between the cell cycle and signaling pathways described above are well defined, the implementation of the cell cycle response to nutrient changes was more challenging due to the complexity of interaction. In the end, we settled for the simplest, most straightforward implementation we could think of. We incorporated nutrient quality as a global parameter that modifies the rate of all protein production reactions equally. The model dynamics scaled appropriately with nutrient quality, i.e. poorer nutritional conditions caused slower accumulation of regulatory proteins leading to slower proliferation, while richer nutrition enhanced cell cycle progression (Fig. 8). This behavior has been observed in multiple experiments (Barberis*et al*. 2007, Boender *et al*. 2009, Gutteridge *et al*. 2010, Jewett *et al*. 2013). Thus, the model is suitable to be extended with details about metabolism and the regulation of cell cycle progression by nutritional cues and quantities. To that end, the respective model parameters, here kinetic parameters *k_p_* of protein production, should be linked to or completely substituted by the output of the metabolic network extension. This would, however, not cover direct effects from signaling pathways that communicate nutritional information such as PKA. Such signaling must be integrated in appropriate fashion.

The presented model also has a number of other properties that will make it useful above the case scenarios for which we have analyzed it here. First, the model comprises a system of only ODEs without any additional algebraic, stochastic or Boolean-like equations, thus remains quite manageable and comprehensible. It is formulated in SBML and it complies with current modeling standards. This is an important aspect to mention for it ensures model reusability. Our model can easily be integrated with any SBML-compliant ODE integrator offering ease of use without risking critical behavior. The model represents realistic orders of magnitude for protein amounts instead of frequently used arbitrary values, thus it can be compared to experimental data and can be integrated with other models employing realistic molecule numbers or concentrations.

Driving the concept of integrating different cellular networks forward, cellular dynamics upon cell cycle progression, development, external stimulation, feeding or other causes of change are extremely complex since, loosely spoken, everything is connected to everything. Given a eukaryotic organism such as yeast with about 6000 genes (Goffeau *et al*. 1996), this complexity and its temporal dimension cannot be fully presented in computational models, yet. However, to get a deeper understanding of cellular regulation, it is crucial to combine the better and better understood building blocks of cell behavior in a sensible way. The presented model with its capabilities is now a prime candidate to serve as a scaffold to integrate the interaction of cell cycle with further signaling and regulation pathways. In the long run, models of that type are required to create preliminary and, later, more mature versions of more complex and comprehensive models for eukaryotic cells, as has been demonstrated already for bacterial cells (Browning and Shuler 2001, Karr *et al*. 2012, Tomita *et al*. 1999). While current versions of cell cycle models or models for signaling pathways or other cellular networks such as metabolism may not be sufficient to describe every interesting aspect precisely, they can serve to test hypotheses and different concepts, e.g. about the action of drugs or the effect of gene expression modifications. This way, a critical discussion of concepts and models that link different types of cellular networks will broaden our understanding of cellular regulation and pave the way for more global descriptions that provide experimentally testable predictions.

## Supplementary Material

foac026_Supplemental_Files
