# Supplementary material for: A yeast cell cycle model integrating stress, signaling, and physiology
Source: FEMS Yeast Res. 2022 May 25;22(1):foac026. doi: 10.1093/femsyr/foac026 (PMC9246278; doi:10.1093/femsyr/foac026)
Supplement: foac026_Supplemental_Files [file foac026_supplemental_files.zip › Supplementary_material.pdf]

Supplementary information

A yeast cell cycle model integrating stress, signaling and physiology

Stephan O. Adler, Thomas W. Spiesser, Friedemann Uschner, Ulrike Münzner, Jens Hahn, Marcus Krantz and Edda Klipp

Table S1: Rate equations

* Clb5 *

$v_1 = nutrition\,factor * \frac{k_{pClb5} * MBF}{K_{pClb5} + MBF} * \frac{1}{(1 + k_{IClb5Hog1} * Hog1_{pp})}$

$v_2 = k_{dClb5} * Clb5$

$v_3 = k_{dClb5-APC} * Clb5 * APC$

$v_4 = k_{cfClb5Sic1} * Clb5 * Sic1$

$v_5 = k_{cfClb5Sic1_{Hp}} * Clb5 * Sic1_{Hp}$

$v_6 = k_{cdClb5Sic1} * Clb5Sic1$

$v_7 = k_{cdClb5Sic1_{Hp}} * Clb5Sic1_{Hp}$

$v_8 = Clb5Sic1 * \left(\frac{k_{ppCln2-Sic1} * Cln2^{n1}}{K_{ppCln2-Sic1}^{n1} + Cln2^{n1}} + \frac{k_{ppClb5-Sic1} * Clb5^{n1}}{K_{ppClb5-Sic1}^{n1} + Clb5^{n1}}\right)$

$v_9 = k_{dClb5Sic1} * Clb5Sic1$

* Cln2 *

$v_{10} = nutrition\,factor * \frac{k_{pCln2} * SBF}{K_{pCln2} + SBF} * \frac{1}{(1 + k_{ICln2Hog1} * Hog1_{pp})}$

$v_{11} = k_{dCln2} * Cln2$

$v_{12} = k_{cfCln2Far1_p} * Cln2 * Far1_p$

$v_{13} = k_{cdCln2Far1_p} * Cln2Far1_p$

$v_{14} = k_{ppCln2Far1_p} * Cln2Far1_p * Cln2$

$v_{15} = k_{dCln2Far1_p} * Cln2Far1_p$

* Cln3 *

$v_{16} = nutrition\,factor * k_{pCln3}$

$v_{17} = k_{dCln3} * Cln3$

$v_{18} = k_{cfCln3Far1_p} * Cln3 * Far1_p$

$v_{19} = k_{cdCln3Far1_p} * Cln3Far1_p$

$v_{20} = k_{ppCln2Far1_p} * Cln3Far1_p * Cln2$

$v_{21} = k_{dCln3Far1_p} * Cln3Far1_p$

* Clb3 *

$v_{22} = nutrition\,factor * \frac{k_{pClb3} * Clb5^{nClb3}}{K_{pClb3}^{nClb3} + Clb5^{nClb3}}$

$v_{23} = k_{dClb3} * Clb3$

$v_{24} = k_{dClb3-APC} * Clb3 * APC$

$v_{25} = k_{cfClb3Sic1} * Clb3 * Sic1$

$v_{26} = k_{cdClb3Sic1} * Clb3Sic1$

$v_{27} = k_{dClb3Sic1} * Clb3Sic1$

* SBF *

$$v_{28} = SBFWhi5 * \left(\frac{k_{ppCln3-Whi5} * Cln3}{K_{ppCln3-Whi5} + Cln3} + \frac{k_{ppCln2-Whi5} * Cln2^{nSBF}}{K_{ppCln2-Whi5}^{nSBF} + Cln2^{nSBF}}\right)$$

$$v_{29} = k_{cfSBFWhi5} * SBF * Whi5$$

$$v_{30} = k_{ppSBF-Clb2} * SBF * Clb2$$

$$v_{31} = k_{dpSBF} * SBF_p * Cdc14_p$$

* Whi5 *

$$v_{32} = nutrition\ factor * k_{pWhi5}$$

$$v_{33} = k_{dWhi5} * Whi5$$

$$v_{34} = Whi5\left(k_{ppCln2-Whi5} * Cln2 + k_{ppCln3-Whi5} * Cln3\right)$$

$$v_{35} = k_{dpWhi5} * Whi5_p * Cdc14_p$$

$$v_{36} = k_{dWhi5_p} * Whi5_p$$

* MBF *

$$v_{37} = nutrition\ factor * \frac{k_{pMBF} * Cln2^{n1}}{K_{pMBF}^{n1} + Cln2^{n1}}$$

$$v_{38} = k_{dMBF} * MBF$$

* Sic1 *

$$v_{39} = nutrition\ factor * \frac{k_{pSic1} * Swi5}{K_{pSic1} + Swi5}$$

$$v_{40} = k_{dSic1} * Sic1$$

$$v_{41} = Sic1 * \left(\frac{k_{ppCln2-Sic1} * Cln2^{n1}}{K_{ppCln2-Sic1}^{n1} + Cln2^{n1}} + \frac{k_{ppClb5-Sic1} * Clb5^{n1}}{K_{ppClb5-Sic1}^{n1} + Clb5^{n1}}\right)$$

$$v_{42} = k_{dSic1_p} * Sic1_p$$

$$v_{43} = k_{ppSic1-Hog1} * Sic1 * Hog1_{pp}$$

$$v_{44} = k_{ppClb5Sic1-Hog1} * Clb5Sic1 * Hog1_{pp}$$

$$v_{45} = k_{dpSic1_{Hp}} * Sic1_{Hp}$$

$$v_{46} = k_{dpClb5Sic1_{Hp}} * Clb5Sic1_{Hp}$$

* Far1 *

$$v_{47} = nutrition\ factor * (k_{pFar1} * Fus3 + k_{pbasalFar1})$$

$$v_{48} = k_{dFar1} * Far1$$

$$v_{49} = k_{ddFar1} * Far1 * Cln2$$

$$v_{50} = k_{ppFar1} * Far1 * Fus3$$

$$v_{51} = k_{dpFar1_p} * Far1_p$$

$$v_{52} = k_{dFar1_p} * Far1_p$$

$$v_{53} = k_{ddFar1_p} * Far1_p * Cln2$$

* Mcm1 *

$$v_{54} = nutrition\ factor * \frac{v0_{Mcm1} * Clb3^{nMcm1}}{V0_{Mcm1}^{nMcm1} + Clb3^{nMcm1}}$$

$$v_{55} = nutrition\ factor * \frac{k_{pMcm1} * Clb2^{nMcm1}}{K_{pMcm1}^{nMcm1} + Clb2^{nMcm1}}$$

$$v_{56} = k_{dMcm1} * Mcm1$$

* Clb2 *

$v_{57} = nutrition\ factor * \frac{k_{pClb2} * Mcm1}{K_{pClb2} + Mcm1} * \frac{1}{(1 + k_{IClb2Hog1} * Hog1_{pp})}$

$v_{58} = k_{dClb2} * Clb2$

$v_{59} = k_{dClb2-APC} * Clb2 * APC$

$v_{60} = k_{ppClb2} * Clb2 * Swe1$

$v_{61} = k_{dpClb2_p} * Clb2_p * Mih1$

$v_{62} = k_{cfClb2Sic1} * Clb2 * Sic1$

$v_{63} = k_{cdClb2Sic1} * Clb2Sic1$

$v_{64} = k_{dClb2Sic1} * Clb2Sic1$

$v_{65} = k_{dClb2_p} * Clb2_p$

* Swe1 *

$v_{66} = nutrition\ factor * k_{pSwe1}$

$v_{67} = k_{dSwe1} * Swe1$

$v_{68} = k_{ppSwe1Clb2} * Clb2 * Swe1$

$v_{69} = k_{dSwe1_p} * Swe1_p$

$v_{70} = k_{ppSwe1Hsl1} * Swe1 * \frac{1}{(1 + k_{ISwe1Hog1} * Hog1_{pp})}$

* RENT/Cdc14 *

$v_{71} = k_{ppCdc14-Clb2} * Clb2 * Cdc14$

$v_{72} = k_{ppCdc14-MEN} * Cdc14_p * Cdc14$

$v_{73} = k_{aCdc14-APC} * APC * Cdc14$

$v_{74} = k_{iCdc14} * Cdc14_p$

* APC *

$v_{75} = nutrition\ factor * \frac{k_{pAPC} * Mcm1}{K_{pAPC} + Mcm1}$

$v_{76} = k_{aAPC-Cdc14} * Cdc14_p * APC_p$

$v_{77} = k_{ppAPC-Clb2} * APC * Clb2$

$v_{78} = k_{ppAPC-Clb5} * APC * Clb5$

$v_{79} = k_{dAPC} * APC$

$v_{80} = k_{dAPC_p} * APC_p$

* Swi5 *

$v_{81} = nutrition\ factor * \frac{k_{pSwi5} * Mcm1}{K_{pSwi5} + Mcm1}$

$v_{82} = k_{dpSwi5-Cdc14} * Cdc14_p * Swi5_p$

$v_{83} = k_{dSwi5} * Swi5$

$v_{84} = k_{ppSwi5-Clb2} * Swi5 * Clb2$

$v_{85} = k_{ppSwi5-Clb5} * Swi5 * Clb5$

$v_{86} = k_{dSwi5_p} * Swi5_p$

* Mih1 *

$v_{87} = nutrition\ factor * k_{pMih1}$

$v_{88} = k_{dMih1} * Mih1$

ODEs:

$$\dot{Cln3} = v_{16} - v_{17} - v_{18} + v_{19} + v_{20}$$
$$\dot{Cln3Far1_p} = v_{18} - v_{19} - v_{20} - v_{21}$$
$$\dot{Cln2} = v_{10} - v_{11} - v_{12} + v_{13} + v_{14}$$
$$\dot{Cln2Far1_p} = v_{12} - v_{13} - v_{14} - v_{15}$$
$$\dot{SBF} = v_{28} - v_{29} - v_{30} + v_{31}$$
$$\dot{SBF_p} = v_{30} - v_{31}$$
$$\dot{SBFWhi5} = -v_{28} + v_{29}$$
$$\dot{Whi5} = -v_{29} + v_{32} - v_{33} - v_{34} + v_{35}$$
$$\dot{Whi5_p} = v_{28} + v_{34} - v_{35} - v_{36}$$
$$\dot{MBF} = v_{37} - v_{38}$$
$$\dot{Clb5} = v_1 - v_2 - v_3 - v_4 - v_5 + v_6 + v_7 + v_8$$
$$\dot{Sic1} = -v_4 + v_6 - v_{25} + v_{26} + v_{39} - v_{40} - v_{41} - v_{43} + v_{45} - v_{62} + v_{63}$$
$$\dot{Sic1_p} = v_8 + v_{41} - v_{42}$$
$$\dot{Clb5Sic1} = v_4 - v_6 - v_8 - v_9 - v_{44} + v_{46}$$
$$\dot{Clb3} = v_{22} - v_{23} - v_{24} - v_{25} + v_{26}$$
$$\dot{Clb3Sic1} = v_{25} - v_{26} - v_{27}$$
$$\dot{Clb2} = v_{57} - v_{58} - v_{59} - v_{60} + v_{61} - v_{62} + v_{63}$$
$$\dot{Clb2Sic1} = v_{62} - v_{63} - v_{64}$$
$$\dot{Clb2_p} = v_{60} - v_{61} - v_{65}$$
$$\dot{Swe1} = v_{66} - v_{67} - v_{68} - v_{70}$$
$$\dot{Swe1_p} = v_{68} - v_{69} + v_{70}$$
$$\dot{Mcm1} = v_{54} + v_{55} - v_{56}$$
$$\dot{APC} = v_{75} + v_{76} - v_{77} - v_{78} - v_{79}$$
$$\dot{APC_p} = -v_{76} + v_{77} + v_{78} - v_{80}$$
$$\dot{Cdc14} = -v_{71} - v_{72} - v_{73} + v_{74}$$
$$\dot{Cdc14_p} = v_{71} + v_{72} + v_{73} - v_{74}$$
$$\dot{Mih1} = v_{87} - v_{88}$$
$$\dot{Swi5} = v_{81} + v_{82} - v_{83} - v_{84} - v_{85}$$
$$\dot{Swi5_p} = -v_{82} + v_{84} + v_{85} - v_{86}$$
$$\dot{Sic1_{Hp}} = -v_5 + v_7 + v_{43} - v_{45}$$
$$\dot{Clb5Sic1_{Hp}} = v_5 - v_7 + v_{44} - v_{46}$$
$$\dot{Far1} = v_{47} - v_{48} - v_{49} - v_{50} + v_{51}$$
$$\dot{Far1_p} = -v_{12} + v_{13} - v_{18} + v_{19} + v_{50} - v_{51} - v_{52} - v_{53}$$

Table S2: Parameter values

k_{pClb5} = 1.87453 (particles * s^{-1})
K_{pClb5} = 1.0 (particles)
k_{pCln2} = 28.0496 (particles * s^{-1})
K_{pCln2} = 22.025 (particles)
k_{pCln3} = 0.01405 (particles * s^{-1})
k_{pClb3} = 0.5116 (particles * s^{-1})
K_{pClb3} = 1.0 (particles)

$k_{pMBF} = 3.0$ (particles * s^{-1})
$K_{pMBF} = 1.0$ (particles)
$k_{pSic1} = 3.16963$ (particles * s^{-1})
$K_{pSic1} = 15.028$ (particles)
$k_{pFar1} = 2.99897$ (s^{-1})
$k_{pbasalFar1} = 0.14066$ (particles * s^{-1})
$k_{pWhi5} = 0.16533$ (particles * s^{-1})
$k_{dClb5} = 0.187825$ (s^{-1})
$k_{dClb2Sic1} = 0.5$ (s^{-1})
$k_{dClb3Sic1} = 0.01$ (s^{-1})
$k_{dClb5Sic1} = 0.1$ (s^{-1})
$k_{dCln2} = 0.2267$ (s^{-1})
$k_{dCln2Far1p} = 0.1$ (s^{-1})
$k_{dCln3Far1p} = 0.0005$ (s^{-1})
$k_{dCln3} = 0.01$ (s^{-1})
$k_{dClb3} = 0.005$ (s^{-1})
$k_{dClb3\text{-}APC} = 0.05$ (particles^{-1} * s^{-1})
$k_{dClb5\text{-}APC} = 1.001$ (particles^{-1} * s^{-1})
$k_{dFar1} = 0.02$ (s^{-1})
$k_{ddFar1} = 0.0033$ (particles^{-1} * s^{-1})
$k_{ddFar1p} = 0.05$ (particles^{-1} * s^{-1})
$k_{ppFar1} = 10.0$ (particles^{-1} * s^{-1})
$k_{dpFar1p} = 0.05$ (s^{-1})
$k_{dFar1p} = 0.1$ (s^{-1})
$k_{dMBF} = 0.2527$ (s^{-1})
$k_{dSic1} = 0.1$ (s^{-1})
$k_{dSic1p} = 0.1$ (s^{-1})
$k_{dWhi5} = 0.01$ (s^{-1})
$k_{dWhi5p} = 0.01$ (s^{-1})
$k_{cfSBFWhi5} = 3.0$ (particles^{-1} * s^{-1})
$k_{cfClb2Sic1} = 15.0$ (particles^{-1} * s^{-1})
$k_{cdClb2Sic1} = 1.0$ (s^{-1})
$k_{cfClb3Sic1} = 30.0$ (particles^{-1} * s^{-1})
$k_{cdClb3Sic1} = 1.0$ (s^{-1})
$k_{cfClb5Sic1} = 30.0$ (particles^{-1} * s^{-1})
$k_{cdClb5Sic1} = 1.0$ (s^{-1})
$k_{cfClb5Sic1Hp} = 600.0$ (particles^{-1} * s^{-1})
$k_{cdClb5Sic1Hp} = 2.0$ (s^{-1})
$k_{cfCln2Far1p} = 6.0$ (particles^{-1} * s^{-1})
$k_{cdCln2Far1p} = 0.5$ (s^{-1})
$k_{cfCln3Far1p} = 6.0$ (particles^{-1} * s^{-1})
$k_{cdCln3Far1p} = 0.5$ (s^{-1})
$k_{ppCln3\text{-}Whi5} = 0.403202$ (particles^{-1} * s^{-1})
$K_{ppCln3\text{-}Whi5} = 85.0316$ (particles)
$k_{ppCln2\text{-}Whi5} = 2.0105$ (particles^{-1} * s^{-1})
$K_{ppCln2\text{-}Whi5} = 1.001$ (particles)
$k_{ppCln2\text{-}Sic1} = 0.43$ (s^{-1})
$K_{ppCln2\text{-}Sic1} = 40.0$ (particles)
$k_{ppClb5\text{-}Sic1} = 21.0$ (s^{-1})
$K_{ppClb5\text{-}Sic1} = 24.99$ (particles)

$k_{ppCln2Far1p}$ = 36 (particles^{-1} * s^{-1})
$k_{ppSBF\text{-}Clb2}$ = 4.05868 (particles^{-1} * s^{-1})
k_{dpSBF} = 0.5 (particles^{-1} * s^{-1})
k_{dpWhi5} = 1.0 (particles^{-1} * s^{-1})
$n1$ = 6.0 (dimensionless)
$nSBF$ = 4.0 (dimensionless)
$nClb3$ = 3.0 (dimensionless)

v_{0Mcm1} = 40.0509 (particles * s^{-1})
V_{0Mcm1} = 20.404 (particles)
k_{dMcm1} = 0.30555 (s^{-1})
k_{pMcm1} = 60.0 (particles * s^{-1})
K_{pMcm1} = 2.5 (particles)
n_{Mcm1} = 3.0 (dimensionless)

k_{pClb2} = 121.56876 (particles * s^{-1})
K_{pClb2} = 5000.0 (particles)
k_{dClb2} = 0.025 (s^{-1})
k_{dClb2p} = 0.025 (s^{-1})
k_{ppClb2} = 350.268 (particles^{-1} * s^{-1})
$k_{dpClb2p}$ = 15.7843 (particles^{-1} * s^{-1})

$k_{ppCdc14\text{-}Clb2}$ = 0.00700465 (particles^{-1} * s^{-1})
$k_{ppCdc14\text{-}MEN}$ = 0.00001 (particles^{-1} * s^{-1})
$k_{aCdc14\text{-}APC}$ = 0.5028 (particles^{-1} * s^{-1})
k_{iCdc14} = 5.02922 (s^{-1})

$k_{dClb2\text{-}APC}$ = 0.604 (particles^{-1} * s^{-1})
k_{pAPC} = 671.03704 (particles * s^{-1})
K_{pAPC} = 94288.25 (particles)
$k_{aAPC\text{-}Cdc14}$ = 3.0 (particles^{-1} * s^{-1})
$k_{ppAPC\text{-}Clb2}$ = 1.00935 (particles^{-1} * s^{-1})
$k_{ppAPC\text{-}Clb5}$ = 100.0 (particles^{-1} * s^{-1})
k_{dAPC} = 0.22 (s^{-1})
k_{dAPCp} = 0.5 (s^{-1})

k_{pSwe1} = 0.56044 (particles * s^{-1})
k_{dSwe1} = 0.1 (s^{-1})
$k_{ppSwe1Clb2}$ = 0.0995 (particles^{-1} * s^{-1})
k_{dSwe1p} = 0.1 (s^{-1})
$k_{ppSwe1Hls1}$ = 1.596 (s^{-1})

$k_{ppSwi5\text{-}Clb2}$ = 99.8 (particles^{-1} * s^{-1})
$k_{ppSwi5\text{-}Clb5}$ = 0.5005 (particles^{-1} * s^{-1})
$k_{dpSwi5\text{-}Cdc14}$ = 50.0 (particles^{-1} * s^{-1})
k_{pSwi5} = 2.39201 (particles * s^{-1})
K_{pSwi5} = 1000.0 (particles)
k_{dSwi5} = 0.013 (s^{-1})
k_{dSwi5p} = 0.013 (s^{-1})

$k_{pMih1} = 0.214$ (particles * s^{-1})
$k_{dMih1} = 0.1$ (s^{-1})

Hog1-parameters:

$k_{ICln2\,Hog1} = 20.029$ (particles^{-1})
$k_{IClb5\,Hog1} = 1.0$ (particles^{-1})
$k_{ppSic1\text{-}Hog1} = 10.0$ (particles^{-1} * s^{-1})
$k_{ppClb5\,Sic1\text{-}Hog1} = 10.0$ (particles^{-1} * s^{-1})
$k_{dpSic1\mathrm{Hp}} = 1.5115$ (s^{-1})
$k_{dpClb5\,Sic1\mathrm{Hp}} = 0.2$ (s^{-1})
$k_{IClb2\,Hog1} = 0.0001$ (particles^{-1})
$k_{ISwe1\,Hog1} = 100.1$ (particles^{-1})

$nutrition\ factor = 1.0$ (dimensionless)

Table S3: Average protein amounts

The presented data is derived from the PaxDB (https://www.pax-db.org/) with the assumption of a total of 50 million protein molecules overall in the samples. The given numbers in rows "Data Base" and "Simulation" are molecule numbers. The relative deviations are visualized in Figure S1.

	APC	Cdc14	Clb2	Clb3	Clb5	Cln2	Cln3	Far1	Sic1	Swe1	Swi5	Whi5	Mcm1	Mih1	SBF	MBF
Data Base	82	3625	580	424	194	650	71	223	400	665	328	940	3995	102	327	329
Simulation	50	3760	394	431	222	306	70	206	422	280	389	1023	2475	107	350	213
Ratio	0.61	1.04	0.68	1.02	1.15	0.47	0.99	0.93	1.06	0.42	1.19	1.09	0.62	1.05	1.07	0.65
rel. dev.	-0.39	0.04	-0.32	0.02	0.15	-0.53	-0.01	-0.07	0.06	-0.58	0.19	0.09	-0.38	0.05	0.07	-0.35

Figure S1: Relative protein abundances

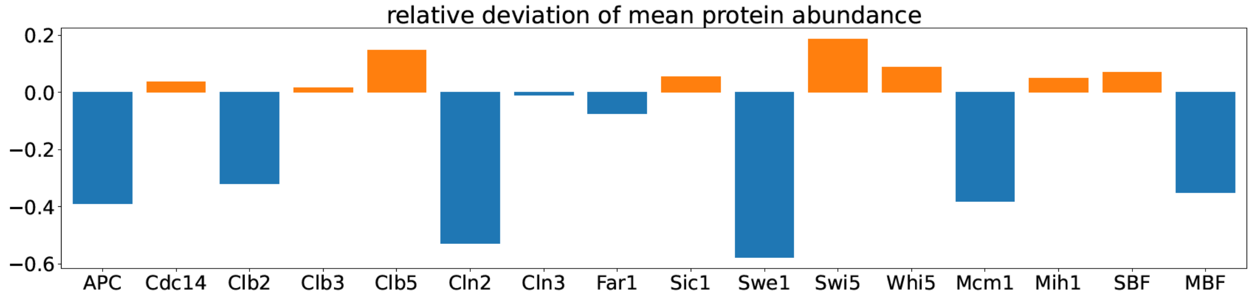

Relative deviations as given in Table 3.

Figure S2: Sensitivity

The following (next page) Figure shows the influence of parameters on the mean cell cycle duration in the simulations. The parameters are sorted by the extent of their influence beginning with the highest impact. The ‘+’ column shows all changes due to a 10% increase of the given parameter and the ‘-’ column for a 10% decrease.

	+10%	-10%
kd_APC	-0.023	0.029
kpp_Cln3_Whi5	-0.012	0.015
kp_Cln3	-0.012	0.015
kd_Cln3	0.013	-0.013
kd_MBF	-0.01	0.013
kd_Cln2	-0.0084	0.012
V0_Mcm1	0.01	-0.011
n_Mcm1	0.01	-0.011
kp_Clb3	-0.0079	0.0093
n_SBF	0.0075	-0.0087
kp_Cln2	-0.0078	0.0078
kp_Whi5	0.0072	-0.0076
Kpp_Cln3_Whi5	0.0072	-0.0075
kd_APC_p	0.007	-0.0076
kd_Mcm1	-0.0053	0.0087
Kpp_Cln2_Sic1	0.0055	-0.0078
Kp_Cln2	0.0064	-0.007
kp_APC	-0.0058	0.0072
Kp_APC	0.0065	-0.0063
ka_APC_Cdc14	-0.0059	0.0067
kcf_SBF_Whi5	0.0057	-0.006
kpp_SBF_Clb2	-0.0051	0.0063
kd_Clb2_APC	-0.0049	0.0061
kdp_Clb2	-0.0051	0.0059
kp_Mih1	-0.0051	0.0059
K_MBF	-0.0051	0.0059
kpp_Clb2	0.0053	-0.0056
kp_Swe1	0.0053	-0.0056
kd_Mih1	0.0053	-0.0056
Kpp_Cln2_Whi5	0.005	-0.0054
kd_Clb5	-0.0047	0.0056
kd_Sic1	-0.0047	0.0053
kp_Clb5	-0.0048	0.0052
kp_Sic1	0.0049	-0.005
kpp_APC_Clb5	0.0046	-0.0051
v0_Mcm1	-0.0039	0.0043
kd_Swi5	-0.0039	0.0041
kdp_SBF	0.004	-0.0035
kd_Clb5_APC	-0.0035	0.0038
kpp_Swe1_Hls1	-0.0034	0.0038
kp_Swi5	0.0035	-0.0037
Kp_Sic1	-0.0033	0.0038
kdp_Whi5	0.0031	-0.0034
Kp_Swi5	-0.0029	0.0032
kpp_Cln2_Whi5	-0.0027	0.0031
n1	0.0025	-0.0032
Kp_Clb2	0.0025	-0.0024
kd_Whi5p	-0.0024	0.0025
kp_Clb2	-0.002	0.0027
kd_Clb3_Sic1	0.0022	-0.0023
kd_Clb3_APC	0.002	-0.0021
Kp_Mcm1	-0.0016	0.002
kp_Mcm1	-0.0017	0.0018
Kp_Clb5	-0.0015	0.0018
kpp_Swe1_Clb2	-0.0016	0.0017

	+10%	-10%
kp_MBF	0.0016	-0.0017
kd_Whi5	-0.0016	0.0016
kpp_APC_Clb2	0.0016	-0.0015
Kpp_Clb5_Sic1	0.001	-0.0018
ki_Cdc14	-0.00097	0.0018
kpp_Cln2_Sic1	-0.0012	0.0013
kd_Clb5_Sic1	-0.0012	0.0013
kcd_Clb3_Sic1	-0.0011	0.0013
kcd_Clb5_Sic1	0.0012	-0.0012
kcf_Clb5_Sic1	-0.0011	0.0012
kd_Swi5_p	-0.001	0.0011
kcf_Clb3_Sic1	0.001	-0.00097
kd_Clb3	0.00082	-0.00082
Kp_Clb3	0.00068	-0.00075
ka_Cdc14_APC	0.00061	-0.00068
kcf_Clb2_Sic1	0.00055	-0.00061
kpp_Swi5_Clb5	-0.00055	0.00061
kpp_Cdc14_Clb2	0.00061	-0.00014
kcd_Clb2_Sic1	-0.00034	0.00041
kdp_Swi5_Cdc14	-0.00027	0.00041
kpp_Swi5_Clb2	0.00034	-0.00034
kd_Clb2_Sic1	0.00027	-0.00034
kd_Clb2_p	0.00027	-0.00027
kd_Swe1	-0.0002	0.0002
kpp_Clb5_Sic1	-0.0002	0.0002
n_Clb3	-0.00014	0.0002
kd_Clb2	0.00014	-6.8e-05
kpp_Far1	0	0
kdd_Far1	0	0
kd_Cln3_Far1p	0	0
kcd_Cln3_Far1p	0	0
kcd_Cln2_Far1p	0	0
kcf_Cln3_Far1p	0	0
kdd_Far1p	0	0
kcf_Clb5_Sic1_Hp	0	0
kdp_Far1p	0	0
kI_Clb2_Hog1	0	0
kI_Cln2_Hog1	0	0
kI_Clb5_Hog1	0	0
kd_Far1p	0	0
kd_Far1	0	0
kp_basal_Far1	0	0
kp_Far1	0	0
kd_Swe1_p	0	0
kI_Swe1_Hog1	0	0
kdp_Clb5_Sic1_Hp	0	0
kpp_Cdc14_MEN	0	0
kdp_Sic1_Hp	0	0
kpp_Clb5_Sic1_Hog1	0	0
kpp_Sic1_Hog1	0	0
kd_Sic1p	0	0
kd_Cln2_Far1p	0	0
kpp_Cln2_Far1p	0	0
kcf_Cln2_Far1p	0	0
kcd_Clb5_Sic1_Hp	0	0

Figure S3 & S4: Clb5 – Sic1 gradients in phase space

The Figures S3 and S4 illustrate the directions and magnitudes of the gradients in the Clb5 – Sic1 phase space on different scales for different time points within a cell cycle. As the limit cycle of this species pair is mostly 0 for one of the species, Figure S3 is a small scale (0 – 12 particles) representation of Figure S4 (0 – 700 particles). While Fig. S4 demonstrates the systems tendency to drastically pull towards the limit cycle close to the axes, Fig. S3 shows the temporal changes of the gradient's directions close to the limit cycle or on it.

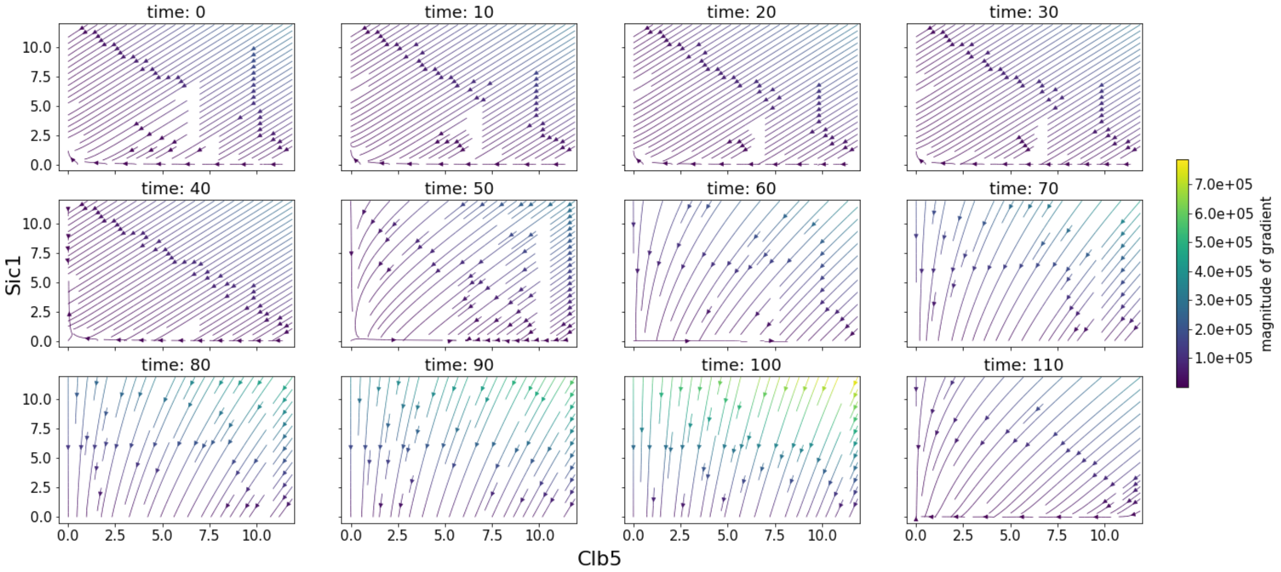

Figure S3: Small scale

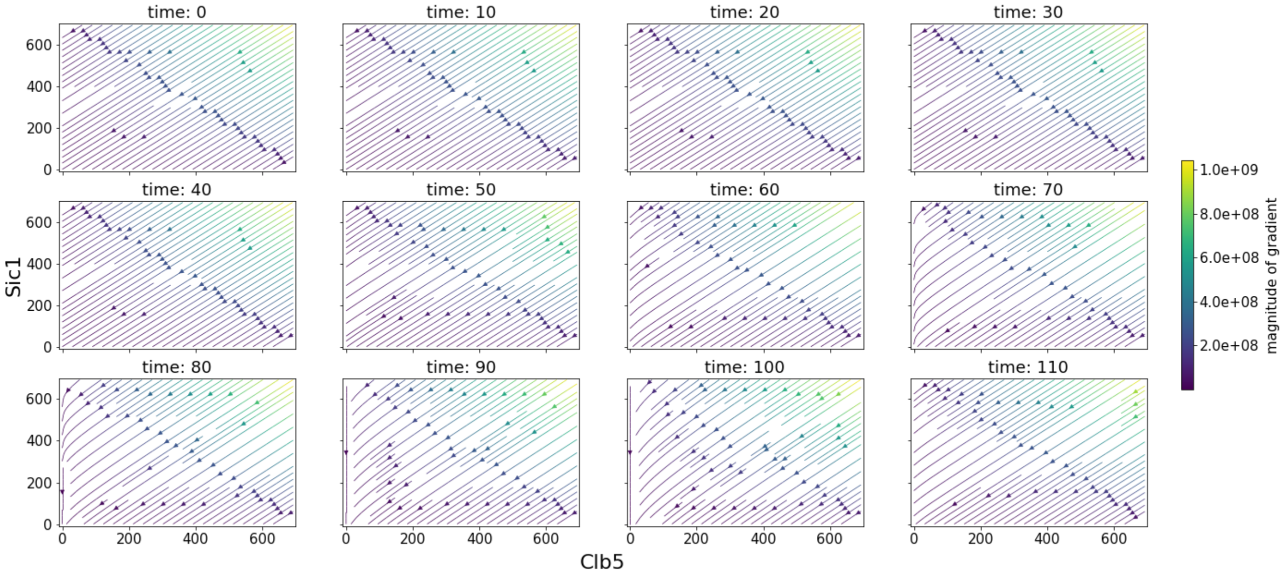

Figure S4: Large scale

Figure S5: UMAP projection of the system in two dimensions

The Figure S5 depicts a UMAP projection fitted to the first cycle in a simulation of 2000min (covering approx. 16.6 cycles). Panel A shows the first cycle alone as the training reference. Panel B illustrates all cycles of the 2000 minute long simulation. Points of similar cell cycle time are grouped closely together, as anticipated, emphasizing the point that all species trajectories are running on a oscillating, stable pattern over time. This is the limit cycle of the system, seen in a two-dimensional projection to the manifold learned by the UMAP.
The colorcode corresponds to the timing within one cell cycle of 122min. The parameters for training are: n_neighbors=45, metric='manhattan' and a min_dist=0.75 in order to separate the points for a better visibility of the 16 cycles. For reproducibility, we used a random_state=42.

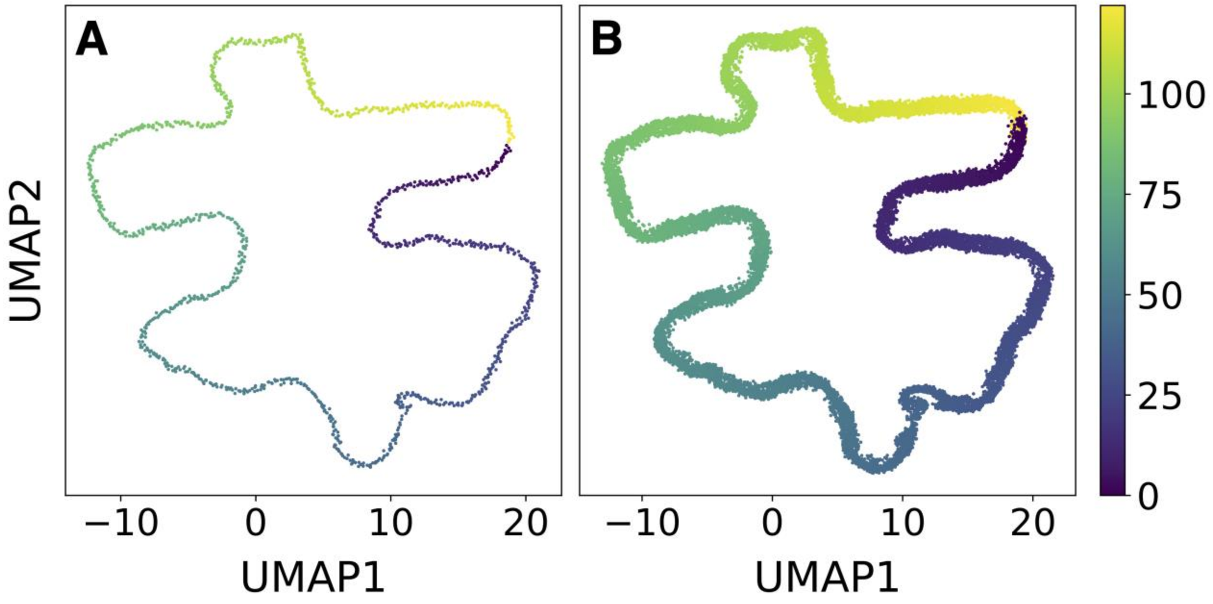

Figure S5: Two-dimensional UMAP of the system

Video S1: Clb5 – Sic1 limit cycle

The video (additional file) displays the temporal evolution of the Clb5 – Sic1 phase space for a wide grid of initial conditions. Within the first few seconds all trajectories from initialized states (single moving dots, blue) move rapidly towards the limit cycle (gray line) on which they keep moving in a repetitive manner. Some of the trajectories, namely the ones that are „out of phase“ compared to the initial conditions of the remaining species in the cell cycle, need one cycle to adjust (Sic1 stays a little lower) but follow the complete cycle afterwards. The rapid initial adjustment is due to the high magnitude of gradients within the system when forced into states far away from the limit cycle (see Figures S3 & S4). After different adjusting times all systems end up on the same trajectory in concert with the whole cycling system, thus producing the limit cycle.
